# Recovery of kidney function in acute kidney injury

**DOI:** 10.1007/s40620-025-02220-w

**Published:** 2025-03-01

**Authors:** Daniel Patschan, Friedrich Stasche, Stefan Erfurt, Igor Matyukhin, Oliver Ritter, Wajima Safi

**Affiliations:** Department of Internal Medicine I - Cardiology, Nephrology and Internal Intensive Care Medicine, University Hospital Brandenburg, Brandenburg Medical School (Theodor Fontane), Hochstraße 29, 14770 Brandenburg an der Havel, Brandenburg Germany

**Keywords:** AKI, AKD, ROKF, KRT, In-hospital mortality

## Abstract

Acute kidney injury (AKI) is associated with a significant burden of mortality worldwide. Each episode of AKI increases the long-term risk of death, especially if there is no recovery or insufficient renal recovery (i.e. restoration of kidney function). This narrative review summarizes relevant studies on the definition and prediction of renal recovery. The following databases were searched for references: PubMed, Web of Science, Cochrane Library, Scopus. The period lasted from 1990 until 2024. The currently available criteria for renal recovery have been identified and discussed. Regarding restoration of kidney function prediction, seven studies on alternative or novel biomarkers have been reviewed. In the context of kidney replacement therapy and renal recovery, findings from four large, prospective randomized studies have been summarized. A standardized definition of renal recovery is presently not available. Specific biomarkers allow for an estimation of the likelihood of renal recovery under certain conditions. According to current knowledge, no dialysis method has been definitively shown to be advantageous for the recovery process.

**Graphical abstract:**

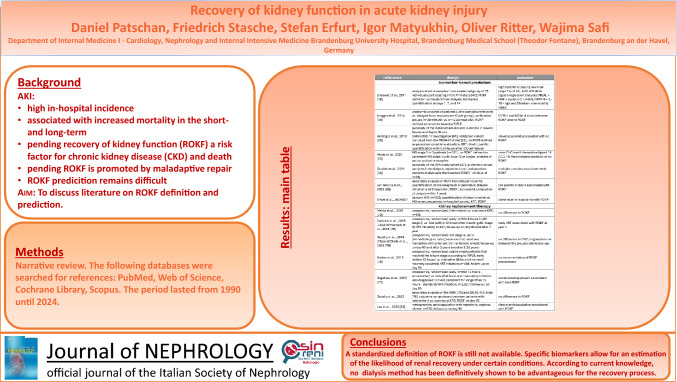

## Introduction

Acute kidney injury (AKI) represents one of the two major nephrological challenges worldwide, alongside chronic kidney disease (CKD). AKI is not a homogeneous entity; rather, it is a clinical condition resulting from a variety of both renal and extrarenal diseases. Ultimately, however, the excretory function of the organ is always impaired. The nosocomial incidence varies worldwide between 5 and 30% [1], with disproportionately higher rates observed in intensive care settings, where it can exceed 50% [2]. AKI is associated with a significantly increased mortality rate, both during the acute phase of the condition and in the subsequent years of life, extending even to the end of life. Residual kidney function disorders lasting beyond a period of 7 days define acute kidney disease (AKD) [3], which, if persistent, transitions into CKD after 3 months. All three entities, AKI, AKD, and CKD, have been identified as risk factors for mortality, with CKD being one of the most potent cardiovascular risk factors overall [4–6]. Therefore, the early detection or prediction of inadequate or absent recovery of kidney function is critically important for prognosis; however, it is rarely achieved in clinical practice. This narrative review will discuss important references on the definition and prediction of restoration of kidney function.

## Methods

The following databases were searched for references: PubMed, Web of Science, Cochrane Library, Scopus. The period lasted from 1990 to 2024. The following search terms were used and combined variably: The following terms were utilized: ‘AKI’, ‘acute kidney injury’, ‘acute renal failure’, ‘recovery of kidney function’, ‘ROKF’, ‘renal restitution’, ‘renal recovery’, ‘kidney recovery’, ‘dialysis’, ‘kidney replacement therapy’, ‘KRT’, ‘renal replacement therapy’, ‘RRT’, ‘biomarker’, ‘AKI biomarker’, ‘stress marker’, ‘damage marker’, ‘functional marker’, ‘clinical determinants’, ‘risk factors’, ‘surrogate markers’, ‘chronic kidney disease’, ‘CKD’, ‘mortality’, ‘cardiovascular risk’, ‘cardiovascular morbidity’, ‘cardiovascular mortality’, ‘cardiovascular death’.

### Acute kidney injury—definition and courses

The definition of AKI has been based on the ‘KDIGO clinical practice guidelines for acute kidney injury’ for 12 years now [7]. The focus is on changes in serum creatinine concentration within a short period of time (48 h or 7 days); urine production is also taken into account. The criteria do not allow early detection of a significant reduction in the glomerular filtration rate (GFR), nor are they in any way specific or even of prognostic significance. The long-needed revision of the criteria is also likely to be so difficult because currently, no individual new biomarker or combination of biomarkers is available that could replace creatinine in all necessary respects while remaining cost-effective. Nevertheless, new criteria have already been discussed. It is conceivable that selected damage biomarkers (measured in serum or urine) could be included in future criteria [8]. The great epidemiological and economic significance of AKI can only be hinted at here. In Central Europe, up to 30% of all hospitalized patients are affected by variable acute renal dysfunction [1], although there are regional differences. The average mortality rate of all affected subjects varies between 10 and 20% [9–11], sometimes exceeding 50% in individuals receiving intensive care [12]. As early as 2013, Lewington and colleagues [13] demonstrated that, from a global perspective, AKI is responsible for more deaths each year than heart failure, diabetes mellitus, breast and prostate cancer combined.

It is not only the acute consequences of the syndrome that determine the prognosis, but also the medium to longer-term consequences. Rewa and Bagshaw [6] documented that each individual episode of AKI significantly increases the risk of death in the following years, even in mild cases according to the RIFLE criteria [14]. A study published in 2013 by Gammelager et al. [15] reported an increase in the hazard ratio for reaching CKD stage 5D (5-year period) to 105 if AKI patients treated with intensive care require dialysis. In a prospective study published by Ikizler et al. [16], hospitalized patients who had not died within 3 months after hospitalization were followed up. Both patients with AKI and those without acute renal dysfunction under inpatient conditions were included. Excretory renal function was determined before hospitalization, 3 months after hospitalization and then annually. Finally, 769 AKI patients were compared with the same number of AKI-negative women and men. Acute kidney injury per se was associated with a significant increase in the adjusted hazard ratio for de novo CKD and CKD progression (3.98 and 2.37, respectively).

Not only the AKI event itself, but above all, residual renal dysfunction after AKI significantly worsens the long-term prognosis. Lee and colleagues [17] analyzed the outcome of dialysis-dependent AKI patients with vs without renal function recovery in a retrospective cohort study. In the second case, there was progression to end-stage kidney disease. Dialysis-dependent AKI patients with renal function recovery showed a 30% lower mortality risk than those without. Kellum and colleagues [18] documented a dramatic association between renal recovery and risk of death during the year after intensive care unit (ICU) admission. Five recovery categories were defined (early sustained, late sustained, relapse recovery, relapse no recovery, and never reversed). In the case of ´never reversed´, less than 50% of the affected AKI patients were still alive after one year, but the late sustained reversal category was also associated with a mortality rate of over 20% after one year. If renal function remains reduced for 7 days after the acute event, AKI will progress to AKD. If no recovery has occurred by the beginning of the 4th month, the criteria for CKD are met [5]. This condition is prognostically highly unfavorable, especially with regard to the cardiovascular disease burden. The dramatic correlation between persistently reduced GFR and cardiovascular disease / death risk was documented almost 20 years ago by Go and colleagues [4]. In the meantime, the consequences of CKD have been characterized even more precisely with regard to various clinical endpoints, as reflected in the latest KDIGO guideline on the subject [5]. However, even if CKD has not yet manifested itself, AKD up to day 90 is extremely unfavorable in terms of prognosis. Sawhney et al. [19] demonstrated mortality rates comparable to those of individuals with AKI after KDIGO [7] up to day 7. At the same time, a larger patient population is included when using the AKD criteria.

In summary, AKI remains a major challenge for both clinical and outpatient medicine. Each episode of AKI increases the morbidity and mortality of those affected, not only in the short term but also in the medium to long term. In terms of morbidity, CKD, with or without the need for dialysis, is a possible consequence, including all associated cardiovascular and other complications. The mortality risk, however, remains elevated over the course of years, even after comparatively mild episodes of AKI.

### Definition of restoration of kidney function (or renal function recovery)

Even today, there are still no standardized criteria for defining renal recovery after AKI.

In a 2014 article [20], Kellum proposed four separate criteria of renal recovery. Three of these had in common the distinction between complete and partial recovery and non-recovery. In 2004, the Acute Dialysis Quality Initiative (ADQI) Group published a report from the second International Consensus Conference [21]. In this document, full renal recovery was defined as a decrease in creatinine concentration to within 50% of the baseline value. Incomplete recovery, on the other hand, was defined as a lower decrease in creatinine while no longer requiring dialysis. Accordingly, non-recovery was present if dialysis was still mandatory. The Acute Tubular Necrosis (ATN) study published in 2008 [22] compared intensive with less intensive kidney replacement therapy (KRT) in AKI individuals in the intensive care unit. Similar, albeit slightly modified, criteria were used in this high-ranking published analysis. Complete and partial recovery were determined on the basis of the creatinine absolute value. The absence of restitution was again present if the patients remained on dialysis. The criteria published by Srisawat and colleagues in 2011 [23] refrain from partial recovery and focus on the need for dialysis as a key differentiating feature between recovery and non-recovery. The RIFLE stages [14] are also taken into account, whereby complete recovery may only be assumed if stage “F” has not been reached. Finally, the fourth group of criteria was taken from the 2012 KDIGO guideline [7]; these were no longer based on serum creatinine but on estimated GFR (eGFR) with graduations of greater than or equal to 60 ml/min for complete recovery and less than 60 ml/min for partial or no recovery (< 90 days: partial recovery, greater than or equal to 90 days: no recovery). Lack of recovery was also postulated if dialysis was still required. The KDIGO criteria have remained valid since 2012, as has the definition of AKI. In his article, Kellum [20] pointed out some of the main limitations of the restoration of kidney function criteria discussed, and emphasized that none of them take the clinical context of AKI into account. Translated, this would mean, for example, that the etiology of AKI is not considered. The highly significant increase in creatinine in the case of transient renal hypoperfusion is, in principle, considered to be of equal value to the decrease in renal function in the case of combined cardiogenic/septic shock. In addition, the definition of full recovery is still arbitrary and exaggerated. Two definitions assume complete restitution if the creatinine concentration is within a certain range above the baseline level. Nevertheless, even low creatinine concentrations reflect significant reductions in GFR. Whether there is actually complete organ recovery remains questionable. However, it is also possible that creatinine is generally limited in the assessment of the recovery process.

In a review also published in 2014, Macedo and colleagues [24] addressed the influence of various kidney replacement procedures on renal recovery. Parameters such as the start of KRT, KRT procedure, the nature of the dialyzer and others were evaluated. Renal recovery was defined as freedom from dialysis. There is no doubt that the lack of need for KRT is a prognostically favorable parameter. Nevertheless, this specification is presumably inadequate, as chronic kidney disease (not requiring dialysis) significantly worsens the long-term cardiovascular prognosis of those affected. The potential problem of every AKI patient is precisely the increase in the risk of CKD.

Forni et al. [25] first emphasized the importance of accurately documenting the baseline level of renal function. This is the only way to decide whether, and to what extent, renal recovery is taking place or not. The aforementioned review plausibly states that renal recovery should be defined as the absence of AKI criteria. Incomplete recovery would therefore exist if a lower AKI stage was reached, whereas complete recovery would exist if any AKI criterion was absent. However, it is essential to know the baseline level. Creatinine-based assessments of renal recovery would be confounded by potential muscle loss during hospitalization. A comparable problem would exist when using the eGFR, which is still predominantly creatinine-based. Finally, Forni et al. state that only the most accurate knowledge of the renal functional reserve immediately before an acute event allows the renal recovery to be determined more or less accurately. However, such data are only available for a small number of patients, for example from individuals undergoing surgery with a high risk of AKI.

In 2019, Xu and colleagues [26] published an article on renal function recovery after AKI in the context of cardiac surgery. The analysis compared 5 distinct recovery criteria. In addition to the criteria already mentioned (ATN study [22], ADQI [3] and KDIGO [7]), the criteria according to Pannu et al. [27] and Bucaloiu and colleagues [28] were taken into account. According to Pannu et al. [27], a drop in serum creatinine to less than 25% above the baseline value defines complete recovery. According to Bucaloiu [28], however, full recovery is assumed when the last eGFR value is greater than or equal to 90% of the initial value. Major adverse events, such as death, new dialysis requirement, progression of CKD, were defined as the combined endpoint at 3 years after the acute event. The recovery rates differed significantly between the criteria: ATN 84.6%; ADQI 82.4%; Pannu 60.49%; KDIGO 68.6%; and Bucaloiu 46.3%. AKI with complete restitution of the kidney function was identified as a major adverse event risk factor when the ATN or ADQI criteria were used. In conclusion, both criteria were found to be only relatively valid, as they appeared to overestimate the recovery rate. This conclusion is particularly plausible because individual episodes of AKI of almost any severity significantly reduce the risk of long-term dialysis and mortality [6].

Finally, in 2020, Duff and Murray [29] proposed a retrospective diagnosis of AKI based on serum creatinine at the time of admission or first presentation. A drop in creatinine of at least 33% (from baseline) within 7 days would define AKI. In addition, the classification allows for an assessment of severity: stage 1 would be present if the drop was 0.66 to 0.49 times baseline, while stages 2 and 3 would be present if the drop was 0.5 to 0.33 times and less than 0.33 times, respectively. In AKD stage 0 all three named stages are absent, however, there may still be indications of organ damage (e.g. positive damage biomarker findings, etc.). These indications allow a further subdivision into categories A, B or C. If there is no further improvement after 7 days, AKI is defined as AKD with or without dialysis. Kidney excretory dysfunction beyond the 90th day, in turn, determines the development of CKD. In the case of AKD, a further distinction is made between the persistent stages 1–3; in stage 3, dialysis may be required. Duff and Murray's concept is commendable as it reflects an attempt to approximate the complex dynamics of the recovery process. The proposal is very far removed from other criteria that assume renal recovery in dialysis-free patients, especially those undergoing intensive care. The authors emphasize the great prognostic significance of residual renal dysfunction (AKD), which significantly increases the lifetime risk of CKD and death [5].

**Fig. 1:**
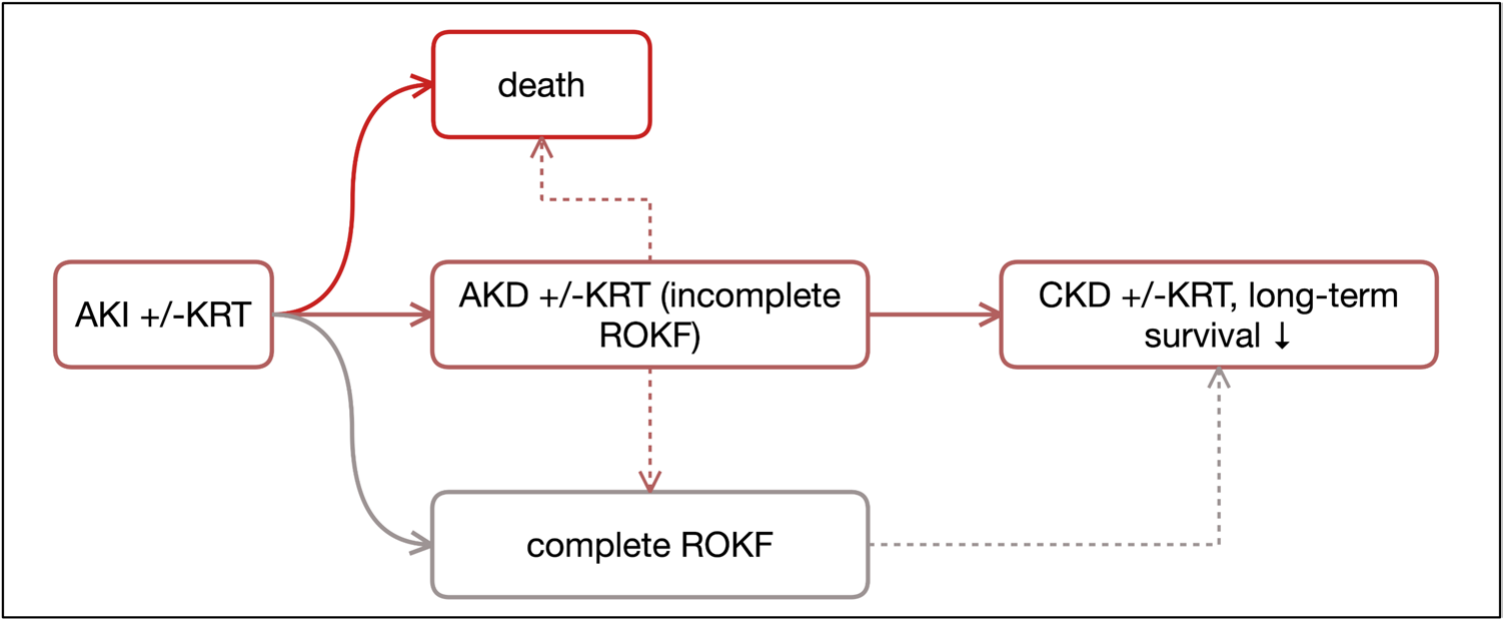
Disease progression courses in AKI. In the short-term course of 7 days, the risk of death of those affected is significantly increased. In principle, recovery of renal function can be incomplete or complete; the criteria proposed to date for this purpose are anything but homogeneous. In principle, restitution does not have to take place. Both patients without recovery and those with incomplete recovery may continue to require dialysis therapy. If the dysfunction persists for more than 7 days, AKD (acute kidney disease) must be assumed. From the 4th month onward, the criteria for CKD are fulfilled

It would be presumptuous at present to formulate a binding definition of renal restitution. In any case, the literature illustrates the effort to take into account the complexity of the renal recovery process and the possible consequences in the event of delayed or incomplete restitution. In both cases (delayed restitution or incomplete restitution > AKD with possible transition to CKD), patients remain at significant risk of premature death as well as cardiovascular complications and the need for dialysis at a later date. As urgently as the definition of AKI itself needs to be revised, optimization and above all standardization of the recovery definition is also necessary. There is hope that the selected candidate molecules from the large number of AKI biomarkers (function, stress, damage) identified to date will be able to improve both diagnostics and prognosis assessment in the future. Figure 1 summarizes the progression pathways of AKI, Table 1 lists the criteria that have been proposed to define recovery. In the following section on restoration of kidney function prediction, the restitution criteria are named in each case.

**Table 1:** Definition criteria of renal recovery (ROKF–recovery of kidney function > renal recovery)

References	Definition
Acute Dialysis Quality Initiative (ADQI) Group Criteria—Bellomo et al. 2004 [21, 23]	Complete ROKF—drop in creatinine concentration to within 50% of the baseline value Incomplete ROKF—modest drop in creatinine without the need for KRT No ROKF—KRT still mandatory
ATN study criteria—Palevsky et al. 2008 [22]	Complete ROKF—serum creatinine level no more than 0.5 mg per deciliter (44 μmol per liter) above the baseline Incomplete ROKF—serum creatinine more than 0.5 mg per deciliter above the baseline value but no KRT dependency No ROKF—KRT dependency
Srisawat et al. 2011 [23]	ROKF—no KRT and no RIFLE ‘F’ nor above No ROKF—KRT or RIFLE ‘F’ or above or death
KDIGO 2012 [7]	Complete ROKF—eGFR ≥ 60 ml/min Incomplete ROKF—eGFR < 60 ml/min No ROKF—KRT dependency
Bucaloiu et al. 2012 [28]	ROKF—eGFR within at least 90% of baseline eGFR occurring within 90 days of AKI
Pannu et al. 2013 [27]	ROKF—post-AKI serum creatinine within 25% of the baseline (pre-hospitalization value) and no KRT dependency
Duff and Murray 2020 [29]	Lowest value within 7 days = 0.66–0.49 or 0.5–0.32 or < 0.32 multiplied by the initial value > stage 1 or 2 or 3 AKI; KRT also defines stage 3; kidney dysfunction persistent for longer than 7 days: AKD, additional grading via biomarkers and serum creatinine

*KRT* kidney replacement therapy, *eGFR* estimated glomerular filtration rate, *AKI* acute kidney injury, *AKD* acute kidney disease

### Restoration of kidney function prediction and associations

#### Biomarker-based prediction of restoration of kidney function

Substantial progress has been made in AKI biomarker research over the last 20 years. The need to identify alternative or new biomarkers arises from the glaring limitations of the marker serum creatinine. In addition to the very late increase in the event of a decrease in GFR, creatinine is characterized above all by a lack of specificity and the absence of any prognostic significance. Finally, the marker does not allow the identification of intrarenal disease processes that are not yet accompanied by a loss of function. Three groups of AKI biomarkers can now be distinguished: functional, damage and stress markers. The most comprehensive group is undoubtedly represented by damage markers. In 2020, Ostermann and colleagues [30] published a congress report on the 23rd Acute Disease Quality Initiative Consensus Conference, in which 23 experts formulated a total of 11 recommendations on the topic of AKI biomarkers. The article summarizes the literature on the three marker groups in tabular form and evaluates the predictive value of individual markers with regard to 5 clinically relevant endpoints: risk assessment, AKI diagnosis and prediction, AKI severity and recovery. In our opinion, the articles now under discussion are of particular importance with regard to prediction of renal recovery.

In 2011, Srisawat and colleagues published a comprehensive study on renal recovery prediction using various biomarkers [23]. The work was carried out as an additive analysis of the ATN study [22]. Urine samples from 76 AKI individuals who were at least temporarily on dialysis were analyzed, whereby renal restitution was defined as freedom from dialysis. The following biomarkers were measured at three time points (days 1, 7 and 14): neutrophil gelatinase-associated lipocalin (NGAL), hepatocyte growth factor (HGF), cystatin C, IL-18 and the neutrophil gelatinase-associated lipocalin/matrix metalloproteinase-9 (MMP-9) ratio. First, cystatin C was significantly higher on day 1 in patients with recovery than in those without recovery. Urine hepatocyte growth factor, on the other hand, was significantly lower in individuals with renal recovery on days 7 and 14. There were further associations for urine NGAL. The most meaningful recovery model resulted from the combined consideration of relative changes in urine biomarkers and various clinical variables (NGAL + HGF + cystatin C + NGAL/MMP-9 + IL-18 + age and Charlson comorbidity index), the receiver-operator characteristics curve was 0.94. This study already points to an important aspect of recovery prediction, the integrative analysis of clinical and laboratory chemical variables.

Another biomarker-based study was published in 2014 [31]. Aregger and colleagues used proteomic analyses from urine samples; each group consisted of 12 critically ill intensive care patients with early or delayed to no recovery. Renal recovery was defined as return to baseline RIFLE. Eight molecules were identified as recovery-predictive: a-1-microglobulin, a-1-antitrypsin, apolipoprotein D, calreticulin, cathepsin D, CD59, insulin-like growth factor-binding protein 7 and neutrophil gelatinase-associated lipocalin. Finally, IGFBP-7 and NGAL were quantified in further analyses of a verification group consisting of 28 individuals with AKI and 12 subjects without acute kidney injury. Both proteins differentiated between patients with early and those with late or no recovery. They also allowed a distinction to be made between AKI + and AKI- individuals. Finally, both factors were predictive of mortality, with an area under the curve (AUC) for NGAL of 0.81. This study also indicates that new AKI biomarkers may not only be of diagnostic but also prognostic value.

The ‘Kidney in Sepsis and Septic Shock’ (Kid-SSS) study conducted in 2018 [32] was designed as a sub-study of the ‘Adrenomedullin and Outcome in Severe Sepsis and Septic Shock (AdrenOSS-1)’ investigation [33]. A validation cohort was included as part of the FROG-ICU trial [34]. The definition of persistent AKI was established as a sustained elevation of serum creatinine by more than 1.5-fold or by at least 0.3 mg/dl (26.5 mmol/l) at day 7 (or at discharge, if earlier), the ongoing requirement for KRT at day 7, or death within 7 days of ICU admission. The primary aim of the Kid-SSS trial was to evaluate the predictive power of proenkephalin A 119–159 (penKid) in septic patients. Proenkephalin A 119–159 acts as a marker of glomerular function, with samples for analysis collected within 24 h of ICU admission. The results indicated that proenkephalin A 119–159 levels were significantly higher in subjects with persistent AKI. In terms of availability and potential impact on daily clinical practice, proenkephalin A 119–159 is already offered for routine diagnostics by some in-hospital laboratories. Its potential role in predicting recovery should not be underestimated, particularly as study participants underwent proenkephalin A 119–159 analysis early in their ICU treatment course.

The primary endpoint of the RUBY study in 2020 [35] was the development of persistent AKI stage 3 (as defined by KDIGO), lasting for 72 h or more. This trial was designed as a multicenter, prospective, observational study, involving ICU-treated patients with AKI stages 2 and 3. Ultimately, 331 out of 364 subjects were included in the endpoint analysis, with 33% reaching the primary endpoint. Serum and urine samples collected at the time of study inclusion were used to quantify various biomarker molecules, including NGAL (urine), KIM-1 (urine), cystatin C (plasma), and C–C motif chemokine ligand 14 (CCL14—urine), among others. Elevated urine C–C motif chemokine ligand 14 emerged as the strongest predictor of the primary endpoint among all tested biomarkers, surpassing KIM-1, cystatin C, and NGAL. The findings suggest that C–C motif chemokine ligand 14 plays a significant role in predicting recovery from AKI, particularly in intensive care settings. However, routine tests for C–C motif chemokine ligand 14 are not yet available, highlighting a gap in clinical practice.

In a 2021 published prospective multicenter study [36], recovery from acute kidney failure was defined as the discontinuation of KRT by day 28 following study inclusion. The authors conducted an analysis of a cohort from the ATN study. The ATN [22] study focused on critically ill patients suffering from severe AKI, which included cases of acute tubular necrosis alongside the failure of one or more non-renal organs. A total of 1124 patients were recruited, with 827 providing at least one serum sample. By day 28, 626 individuals were alive, and 343 no longer required any further KRT. In the current (sub)study, serum samples from both dialysis-dependent (*n* = 34) and dialysis-independent (*n* = 38) patients were utilized for proteomic analysis, encompassing over 1300 proteins. Patients with restoration of kidney function exhibited higher levels of the following proteins: CXCL11, CXCL2/CXCL3, CD86, Wnt-7a, BTK, c-Myc, TIMP-3, CCL5, Ghrelin, PDGF-C, Survivin, CA2, IL-9, EGF, and Neuregulin-1, while displaying lower levels of CXCL16, IL1RL1, Stanniocalcin-1, IL-6, and FGF-23. In summary, the study identified several novel candidates for predicting restoration of kidney function in patients with AKI requiring KRT. Regarding the availability and potential impact on daily clinical practice, it is noteworthy that almost all tested biomarkers are not currently available for routine diagnostics. However, the findings suggest that making decisions about complex processes, such as restoration of kidney function, likely necessitates the simultaneous analysis of multiple markers or marker panels rather than relying on a single protein or peptide.

Von Groote et al. [37] published a secondary analysis of the RICH Trial (“Effect of Regional Citrate Anticoagulation versus Systemic Heparin Anticoagulation During Continuous Kidney Replacement Therapy on Dialysis Filter Life Span and Mortality Among Critically Ill Patients With Acute Kidney Injury-A Randomized Clinical Trial”) [38], in which plasma proenkephalin A 119–159 was quantified at the start of continuous renal replacement therapy (CRRT) and 3 days later. The endpoint was defined as successful completion of KRT, with unsuccessful completion including death within one week of discontinuation of dialysis. Low proenkephalin A 119–159 levels on day 3 were associated with successful KRT weaning, but an even stronger association with dialysis freedom was identified for increased daily urine output. Freedom from dialysis is in principle a recovery indicator, so proenkephalin A 119–159 was obviously suitable as a recovery predictor under the specified study conditions.

A study by our group [39] analyzed the predictive value of serum nostrin in de novo AKI. Nostrin is a protein produced mainly in endothelial cells, which is substantially involved in the regulation of nitric oxide (NO) metabolism [40]. The endothelial-selective knockout of the protein is associated, for example, with significant cardiac insufficiency [41]. The cohort of patients studied has already been described in a previously published paper on the soluble IL-33 receptor (sST-2) [42]. It consisted of 150 patients with initially diagnosed acute kidney injury in hospital. Survival in hospital, dialysis requirement and renal recovery were defined as endpoints. Recovery of kidney function was assumed if the last serum creatinine concentration did not differ from the initial value by more than 50%. All nostrin measurements were taken at the time of initial AKI diagnosis, which was based on KDIGO criteria 1 and 2. Nostrin was predictive for all three endpoints, with comparable performance for NGAL but not for KIM-1. Previously unpublished data from the same cohort show that so-called oxidized high density lipoprotein is also predictive of renal restitution. We expect these data to be published at the beginning of 2025.

A possible limitation of all the biomarker studies cited is, and remains, the definition of recovery. In all cases, it is based either on newly achieved freedom from dialysis or falling below a certain (elevated) creatinine level. The fact that the predictive performance of new biomarkers is measured against criteria based on conventional indicators of kidney function already shows the difficulties in identifying new recovery markers. Even if recovery is assessed via changes in eGFR, the formulas commonly used today (MDRD, CKD-EPI [43]) still take serum creatinine into account. Nevertheless, the continued intensive search for alternatives to creatinine is of course essential.

#### Kidney replacement therapy and restoration of kidney function

As early as 2014, Schneider and Bagshaw published a review article on the effects of KRT on the recovery process after AKI [44]. At the time of publication, only limited systematic, prospective data were available on this specific aspect of AKI therapy. The article evaluated the recovery success in relation to the following categories: decision to undergo KRT at all, timing of KRT initiation, modality of KRT (intermittent versus continuous), KRT dose, nature of the dialysis membrane, and anticoagulation mode. With regard to the modality of KRT, one prospective randomized study was cited at the time [45]. In this multicenter trial, 166 ICU patients were included and randomized to either the intermittent or continuous treatment arm. Under continuous KRT, more patients died in the intensive care unit or in hospital overall. The recovery rates did not differ between the two groups.

Renowned trials have been published since then, particularly with regard to the variable ‘KRT onset’. The studies were published under the leadership of the following authors: Zarbock et al. (ELAIN trial, 2016 [46]), Gaudry et al. (AKIKI trial, 2016 [47]), Barbar et al. (IDEAL-ICU, 2018 [48]) and Bagshaw et al. (STARRT-AKI, 2020 [49]). In the ELAIN trial [46], for example, a prospective, single-center study, 231 ICU patients were dialyzed either within 8 h of diagnosis of AKI (stage 2 according to KDIGO—early) or within 12 h of reaching stage 3 of AKI (late). In the late group, no dialysis was performed in some cases. The early intervention was associated with a significant survival advantage for patients on day 90. The authors extended the follow-up period to one year and the data were published in 2018 [50]. The combined endpoint was defined as persistent renal dysfunction, persistent dialysis requirement and death. In the early intervention group, mortality was lower after one year, while significantly more patients met the criteria for renal recovery. Contrasting data were collected in the AKIKI study [47]. However, the study design differed in some respects. AKI (KDIGO stage 3) patients in the intensive care unit who did not initially present with any mandatory dialysis criteria (e.g. severe hyperkalemia, pulmonary edema, etc.) were included. Dialysis was started either immediately or when at least one mandatory dialysis criterion was diagnosed. Oliguria for up to 72 h was also defined as such. At 60 days, the groups did not differ in terms of mortality. The SALTO analysis [51] from 2023 summarized follow-up data after a median duration of 3.35 years. Initially, overall survival after 3 years was 39.4%, but the mortality risk had decreased from the third month after study inclusion. There were no differences in mortality or CKD progression risk between patients with early versus late dialysis initiation. The SALTO analysis does not explicitly address the issue of recovery; however, the comparable CKD progression risk tends to argue against differences in renal recovery. The results of the IDEAL-ICU study [48] are quite similar to those of the AKIKI trial with regard to the early outcome of those affected. Individuals with septic shock who had reached the failure stage of the RIFLE criteria were included. Early dialysis was given within 12 h, whereas late KRT was initiated after 48 h if there was no renal recovery. At 90 days, there was no difference in mortality between the groups. In 2022, Gaudry et al. finally published a secondary analysis of the AKIKI and IDEAL-ICU studies [52]. Although the primary intention of both studies was to compare early and late dialysis initiation in AKI, this evaluation primarily addressed an essential question of nephrological intensive care medicine, i.e., the comparison of intermittent and continuous KRT. For this purpose, individuals with early dialysis initiation were selected from both studies, and survival on day 60 was defined as the primary endpoint. However, the study evaluated renal recovery up to day 28. On the one hand, the variable “freedom from dialysis” was recorded, on the other hand “kidney recovery” per se. Kidney recovery was defined as “RRT (KRT) discontinuation and spontaneous urine output higher than 1000 mL per 24 h in the absence of diuretic therapy or higher than 2000 mL per 24 h with diuretics”. Again, there was no significant difference in either endpoint between the two treatment groups. Finally, the STARRT-AKI study [49] also compared an early (accelerated) with a prolonged or late (standard) start of KRT. The primary endpoint was defined as mortality on day 90; there were no significant differences. However, a larger proportion of survivors in the accelerated group were still on dialysis at this time. If a conclusion could be drawn from these data, it would not be uniform. In the ELAIN trial, earlier intervention was associated with more frequent restitution, while the situation was diametrically opposed in the STARRT-AKI trial. The other studies did not show different restitution rates. Overall, there is no clear recommendation for an earlier or later start of dialysis if the focus is on restitution. The same probably applies to the parameters of intermittent or continuous dialysis.

Another important determinant of every KRT is the anticoagulation of those treated. In most cases, dialyzed patients receive either heparin (unfractionated or fractionated) or regional citrate anticoagulation. The latter reduces blood clotting mainly in the tubing system and dialysis filters. In 2023, Zhou et al. [53] published a comprehensive meta-analysis on the topic, in which the following relevant endpoints were defined, among others: filter lifespan, all-cause mortality, duration of CRRT, recovery of kidney function. The authors identified 37 randomized controlled studies, with a cumulative number of patients of over 2600. Some patients were also treated with a combination of heparin and prostaglandin I2. Overall, however, no anticoagulation regimen showed a significant survival benefit for those treated, nor were there any significant differences in the frequency of renal recovery. In a retrospective analysis published in 2023, Lau and colleagues [54] compared the outcome of 276 AKI patients in the intensive care unit. Anticoagulation was provided either by heparin or regionally administered citrate, and the combined primary endpoint was defined as mortality and dialysis requirement on day 90 after inclusion. The endpoint was reached significantly less frequently with citrate anticoagulation. However, the authors cite the retrospective design of the study as a limitation, and a possible new dialysis requirement after 90 days was not recorded.

The currently available data do not allow the conclusion that a dedicated KRT procedure or the timing of initiation or use of a specific type of anticoagulation is demonstrably associated with a better recovery prognosis.

### Clinical determinants of renal recovery

Clinical determinants of renal restitution primarily include general patient characteristics as well as comorbidities and medication. It seems plausible to assume that all those conditions that favor maladaptive repair are risk factors for inadequate or absent restitution. As early as 2014, Godin and colleagues [55] summarized risk factors for inadequate or absent recovery, naming older age, arterial hypertension, diabetes mellitus, pre-existing chronic kidney disease and heart failure, proteinuria and others as the main factors or morbidities. At this point, the aforementioned work by Kellum and colleagues in 2017 [18] should be mentioned again. In the study, 5 recovery categories were defined and a total of 16,968 AKI patients were evaluated. The study offers a wide range of insights, as the authors compare different categories of renal recovery and risk factors for achieving or not achieving them. In one of the analyses, risk factors for a lack of restitution in any form are determined, regardless of the form in which recovery occurred (early sustained, late sustained, etc.). Age, pre-existing cardiac disease and the use of vasopressors were identified as risk factors for failure to recover. On the other hand, hypertensive individuals did not show an increased risk of no recovery per se, and the odds ratio was actually reduced (0.89). The study again highlights the significant impact of inadequate restitution on mortality at 365 days, with the odds ratio increasing to 2.68. The specific aspect of vasopressor therapy as a risk factor for AKI and inadequate AKI resolution was addressed in a study published in 2023 by Nishimoto et al. [56]. This was a retrospective study of patients undergoing cardiac surgery; the preoperative use of vasopressors was documented. In the overall cohort of more than 5,000 patients, the postoperative AKI incidence was 6%, and preoperative vasopressor use was independently associated with the occurrence of AKI. In addition, patients treated with vasopressors showed a significantly delayed recovery of renal function within 2 weeks.

Tables 2 and 3 summarize data on the prediction of renal recovery and associations between dialysis modalities and the likelihood/frequency of renal recovery.

**Table 2:** Studies on biomarker-based prediction of recovery of the kidney function

References	Design	Outcome
Srisawat et al. 2011 [23]	Analysis of urine samples from a selected group of 76 individuals participating in the ATN study [22]; recovery of the kidney function: definition as freedom from dialysis; biomarker quantification at days 1, 7, and 14	High cystatin C (day 1), low HGF (days 7 and 14), in logistic regression analysis (NGAL + HGF + cystatin C + NGAL/MMP-9 + IL-18 + age and Charlson comorbidity index) (0.94 (0.87–1.0))
Aregger et al. 2014 [31]	Proteomic analysis of patients (urine samples) with early vs. delayed to no recovery (*n* = 12 per group); verification groups (*n* = 28 with AKI vs. *n* = 12 without AKI); recovery of the kidney function: defined as return to baseline RIFLE	IGFBP-1 (AUC 0.74 (0.61–0.88)) and NGAL (AUC 0.70 (0.56–0.84)) distinct between recovery of the kidney function and no recovery of the kidney function
Hollinger et al. 2018 [32]	Sub-study of the ‘Adrenomedullin and Outcome in Severe Sepsis and Septic Shock (AdrenOSS-1)’ investigation [33], validation cohort recruited from the FROG-ICU trial [34], no recovery of the kidney function is defined as persistent creatinine elevation, KRT, death; penKid quantification within 24 h after ICU admission	Elevated penKid associated with no recovery of the kidney function (adjusted OR for Major Adverse Kidney Events—included persistent AKI—3.3 (1.8–6))
Hoste et al. 2020 [35]	AKI stage 2 or 3 patients (*n* = 331), no recovery of the kidney function is defined as persistent AKI stage 3 until hour 72 or longer, analysis of serum and urine samples	Urine C–C motif chemokine ligand 14 (CCL14) the strongest predictor of no recovery of the kidney function (AUC 0.83 (0.78–0.87))
Daniels et al. 2021 [36]	Sub-study of the ATN study cohort [22], proteomic serum samples from dialysis-dependent and -independent patients (individuals that reached recovery of the kidney function) (*n* = 34 and *n* = 38)	Multiple proteins associated with recovery of the kidney function (many OR provided—see original article)
von Groote et al. 2023 [37]	Secondary analysis of RICH trial [38] participants; quantification of proenkephalin A (penKid) at dialysis initiation and 3 days later; recovery of the kidney function: discotinuation of dialysis within 1 week	Low penKid at day 3 associated with recovery of the kidney function: (sHR 2.35, 95% CI 1.45–3.81, *p* < 0.001)
Erfurt et al. 2024 [39]	De novo AKI (*n* = 150); quantification of serum nostrin at AKI onset; endpoints: in-hospital survival, KRT, recovery of the kidney function	Lower nostrin in patients with recovery of the kidney function: (0.63 (0.53–0.74))

*HGF* hepatocyte growth factor, *NGAL* neutrophil gelatinase-associated lipocalin, *MMP-9* matrix-mettaloproteinase-9, *IL-18* interleukin-18, *RIFLE* risk, injury, failure, loss, end-stage renal disease, *IGFBP-1* insulin-like growth factor binding protein-1, *KRT* kidney replacement therapy, *ICU* intensive care unit

**Table 3:** Studies on associations between the timing of KRT initiation/modality and renal recovery (ROKF)

References	Design	Outcome
Mehta et al. 2001 [45]	Prospective, randomized; intermittent vs. continuous KRT; *n* = 166	No difference in recovery of the kidney function
Zarbock et al. 2016 [46] and Meersch et al. 2018 [50]	Prospective, randomized; early (within 8 h in AKI stage 2) vs. late (within 12 h after reaching AKI stage 3) KRT initiation; *n* = 231, follow-up on day 90 and after 1 year	Early KRT associated with recovery of the kidney function at one year
Gaudry et al. 2016 [47] and Chaibi et al. 2023 [51]	Prospective, randomized; AKI stage 3, early (immediately) vs. late (presence of at least one mandatory KRT criterion) KRT initiation; *n* = 620; follow-up on day 60 and after 3 years (median 3.35 years)	No difference in CKD progression risk between the groups (late follow-up)
Barbar et al. 2018 [48]	Prospective, randomized; septic shock patients that reached the failure stage according to RIFLE; early (within 12 h) vs. late (after 48 h if no renal recovery occurred) KRT initiation; *n* = 488; follow-up on day 90	No documentation of prevalence of recovery of the kidney function
Bagshaw et al. 2020 [49]	Prospective, randomized; early (within 12 h—accelerated) vs. late (if at least one mandatory criterion was diagnosed or if AKI persistent for longer than 72 h—standard) KRT initiation; *n* = 2927; follow-up on day 90	Accelerated approach associated with less recovery of the kidney function
Gaudry et al. 2022 [52]	Secondary analysis of the AKIKI [47] and IDEAL-ICU trials [48]; outcome comparisons between patients with intermittent vs. continuous KRT; recovery of the kidney function on day 28	No difference in recovery of the kidney function
Lau et al. 2023 [54]	Retrospective; anticoagulation with heparin vs. regional citrate; *n* = 276; follow-up on day 90	Citrate anticoagulation associated with recovery of the kidney function

*KRT* kidney replacement therapy, *AKI* acute kidney injury, *CKD* chronic kidney disease, *RIFLE* risk, injury, failure, loss, end-stage renal disease

### Summary and conclusions

Acute kidney injury remains a dramatic problem in inpatient medicine worldwide, with high incidences and a significantly increased risk of death both acutely and in the medium to long term. The long-term increase in mortality is greater if renal dysfunction continues after the acute event. Recognizing the absence of recovery is crucial, not least for socio-economic reasons. There is still no standardized definition of recovery of kidney function. However, newer criteria take into account more intensive residual renal dysfunction, even to a lesser extent. Such seemingly discrete deviations are already associated with a considerable deterioration in the prognosis of those affected. In the meantime, some alternative or new biomarkers have been identified which, under certain conditions, can estimate the chance of renal recovery. The assessment of biomarker performance for estimating recovery is influenced by the fact that, in many cases, recovery detection is still primarily based on changes in serum creatinine levels. According to current knowledge, no specific dialysis procedure can be clearly favored with regard to renal restitution. Clinical determinants of recovery can be identified as factors that may plausibly promote maladaptive repair.

## Data Availability

Not applicable.
